# Longitudinal Characterization of Cytokine Overproduction: A Case Report in Critically Ill COVID-19 Patients With Hyperinflammation in Bronchoalveolar Lavage

**DOI:** 10.3389/fmed.2021.690523

**Published:** 2021-09-07

**Authors:** Zhen Luo, Chengliang Zhu, Zhihui Ruan, Xianghua Cui, Muhammad Adnan Shereen, Pan Pan, Jingtao Huang, Fubing Wang, Hanwen Su, Yuchen Xia, Jianguo Wu

**Affiliations:** ^1^Guangdong Provincial Key Laboratory of Virology, Institute of Medical Microbiology, Jinan University, Guangzhou, China; ^2^Department of Clinical Laboratory, Renmin Hospital of Wuhan University, Wuhan, China; ^3^State Key Laboratory of Virology, College of Life Science, Wuhan University, Wuhan, China; ^4^Department of Laboratory Medicine, Zhongnan Hospital of Wuhan University, Wuhan, China; ^5^State Key Laboratory of Virology, School of Basic Medical Sciences, Wuhan University, Wuhan, China

**Keywords:** SARS-CoV-2, COVID-19, poor outcomes, bronchoalveolar lavage fluid, hyperinflammation, cytokine overproduction

## Abstract

**Objectives:** The longitudinal characterization and risk of poor outcomes related to cytokine overproduction in critical coronavirus disease 2019 (COVID-19) patients with hyperinflammation in bronchoalveolar lavage requires further investigation.

**Methods:** We enrolled two critically ill patients with comorbidities diagnosed with severe acute respiratory syndrome coronavirus 2 (SARS-CoV-2) detected by RT-PCR during hospitalization. Clinical characteristics, longitudinal immunological, and biochemical parameters of each critical COVID-19 case were collected.

**Main Results:** The clinical characteristics and laboratory results of each case demonstrated critical symptoms of COVID-19 with poor outcomes. Both nasopharyngeal swabs and bronchoalveolar lavage fluid (BALF) samples tested positive for SARS-CoV-2. Two patients received targeted treatments against pathogen infection and inflammation in addition to interventional therapies, except for Patient 2, who received an additional artificial liver system treatment. Hyperinflammation with a dominantly high level of IL-6 was observed in BALF samples from both critical cases with decreased T cell populations. High levels of cytokines and pathological parameters were successively maintained in Patient 1, but rapidly reduced at the late treatment stage in Patient 2. The outcome of Patient 1 is death, whereas the outcome of Patient 2 is recovery.

**Conclusions:** This case report suggests that a high risk of poor outcomes was related to a heavily hyperinflammatory milieu in both the blood and lungs of critical COVID-19 patients. The artificial liver intervention on cytokines overproduction might be beneficial for the recovery of critical COVID-19 patients as a reliable therapy that can be coordinated with targeted treatments, which ought to be further tested in adequately designed and powered clinical trials.

## Introduction

Severe acute respiratory syndrome coronavirus 2 (SARS-CoV-2) led to the coronavirus disease 2019 (COVID-19) pandemic; it has a fatality rate of 0.5–1.0% and has led to significant increases in fatalities in those with preexisting comorbidities (1). Upon SARS-CoV-2 infection, the clinical manifestations of most COVID-19 patients are asymptomatic or mild, but some cases would develop acute respiratory distress syndrome (ARDS), multiple organ failure (MOF), and even sudden death (2). During the progression of COVID-19, overproduction of inflammatory cytokines and a dysregulated host immune response, eventually act as a cytokine storm, is considered to be the primary cause of ARDS and MOF in severe and critical patients, thereby contributing to the severity and poor prognosis of the illness (3).

SARS-CoV-2 infection generates a delayed but overactive production of cytokines resembling interleukin-1β (IL-1β), IL-6, IL-8, IL-10, tumor necrosis factor-alpha (TNF-α), and monocyte chemoattractant protein-1, inducing dysregulated innate immune responses (2, 4). In addition, SARS-CoV-2-mediated impairment of lymphoid organs is responsible for lymphopenia, resulting in an impaired adaptive immune response, especially in those with severe infection (5, 6). Since hyperinflammation is closely related to the exacerbation of symptoms in severe and critical COVID-19 patients (7), the relationship between SARS-CoV-2 presence and host hyperinflammation needs to be fully explored.

SARS-CoV-2 preferentially infects type II alveolar cells (AT2) in the lungs as the viral target organs (8, 9), and bronchoalveolar lavage (BAL) samples from COVID-19 patients serve as the optimal cytologic specimens. There is increasing evidence that exuberant plasmacytosis and transcriptional changes of inflammatory genes can be detected in the BAL specimens of COVID-19 patients (10–12), suggesting the presence of hyperinflammation upon SARS-CoV-2 infection in the lungs. Although bronchoalveolar inflammation in COVID-19 patients correlates with the clinical outcomes (13), the dynamic inflammatory response to severe forms of the illness in COVID-19 patients based on the detection of hyperinflammation in BAL specimens is still unclear. In this study, we present the clinical characteristics of two critical COVID-19 patients, as well as the high risk of poor outcomes related to cytokine overproduction during severe COVID-19 treatments with hyperinflammation detected from bronchoalveolar lavage fluid (BALF) samples.

## Methods

### Study Design and Patients

The patients were clinically diagnosed with COVID-19 according to the “pneumonia diagnosis protocol for novel coronavirus infection (trial version 7)” (Released by National Health Commission & State Administration of Traditional Chinese Medicine on March 3, 2020). They were subjected to tests including clinical examination, computed tomography (CT), and real-time reverse-transcription polymerase chain reaction (RT-PCR) for SARS-CoV-2 (Table 1). The patients were admitted to the Renmin Hospital of Wuhan University, Wuhan, China, from January 25 to April 8, 2020. Patient 1 was admitted on February 11, 2020 and transferred into intensive care unit (CCU) on February 18, 2020, while Patient 2 was admitted on February 6, 2020 and transferred into CCU on February 13, 2020 (Table 2).

**Table 1:** Summary of clinical features and laboratory test results of critically ill patients with COVID-19.

	**Patient 1**	**Patient 2**
**Characteristics**		
Age (years)	69	42
Gender	Female	Male
Interval between admission to hospital and symptom onset (days)	8	12
Interval between confirmed test for SARS-CoV-2 and admission to hospital (days)	2	8
CT findings	Blurred borders in both lungs with tuberculosis in upper lobe or apex of the lung	Scattered ground-glass opacities in both lungs
**Symptoms and signs**		
Fever	–	+
Cough	+	+
Nasal congestion	–	–
Asthenia	+	+
Rhinorrhoea	–	–
Poor appetite	–	–
Chest distress	–	–
Dyspnea	+	+
Diarrhea	–	–
Body temperature (°C)	37.1	38.7
**Clinical course**		
Duration of fever (days)	0	12
Duration in CCU	22	55
Duration of hospitalization (days)	29	62
**Laboratory test**		
White blood cell count, × 10^9^/L (Normal range: 3.5–9.5)	11.37	7.12
Neutrophil count, × 10^9^/L (Normal range: 1.8–6.3)	9.52	6.43
Neutrophil ratio, % (Normal range: 40–75)	83.70	90.40
Lymphocyte count, × 10^9^/L (Normal range: 1.1–3.2)	1.00	0.38
Lymphocyte ratio, % (Normal range: 20–50)	8.80	5.30
ATL, U/L (Normal range: 7–40)	36.00	77.00
AST, U/L (Normal range: 13–35)	42.00	64.00
PCR of nasopharyngeal swab	+ Ct = 39	+ Ct = 34.83
PCR of whole blood	–	NA
PCR of sputum	NA	+
PCR of feces	–	+
PCR of urine	+	–
PCR of BALF	+ Ct=34.29	+ Ct=33.8
SARS-CoV-2 IgG, AU/mL (Normal range: < 10)	58.22	159.60
SARS-CoV-2 IgM, AU/mL (Normal range: < 10)	28.17	21.94
ADV DNA	–	–
Boca DNA	–	–
H1N1 RNA	–	–
H3N2 RNA	–	–
HCOV RNA	–	–
HMPV RNA	–	–
HPIV RNA	–	–
HRSV RNA	–	–
HRV RNA	–	–
LPN1 IgM	–	–
CP IgM	–	–
**Treatments**		
Comorbidities	Primary hypertension (Grade III), coagulation disorders, hypokalemia	Secondary hypertension (Grade III), coagulation disorders, hypoalbuminemia
Anti-viral	Oseltamivir, Arbidol	Arbidol, Interferon-alpha, Hydroxychloroquine
Anti-bacterial	Cefperazone-Sulbactam, Moxifloxacin, Piperacillin, Tazobactam, Vancomycin, Polymyxin B, Tigecycline	Cefperazone-Sulbactam, Meropenem, Teicoplanin, Imipenem, Tigecycline, Polymyxin B, Vancomycin, Daptomycin, Linezolid
Anti-fungal	Voriconazole, Micafungin	Voriconazole, Micafungin
Anti-inflammatory	Intravenous immune globulin, Methylprednislone	Intravenous immune globulin, Methylprednislone
Supplemental oxygen	Invasive ventilation	Invasive ventilation
Interventional therapy	Bronchoscopy, bronchoalveolar lavage, Thoracic closed drainage	Li's ALS, Bronchoscopy, bronchoalveolar lavage, Thoracic closed drainage
Outcomes	Died	Survived

*NA, not available; +, positive; –, negative; CT, Computed Tomography; CCU, Cardiovascular Intensive Care Unit; ALT, Alanine aminotransferase; AST, Aspartate aminotransferase; PCR, short for Real-time PCR against SARS-CoV-2 nucleic acid; Ct, Cycle threshold value of SARS-CoV-2 N gene; BALF, bronchoalveolar lavage fluid; ADV, Adenovirus; H1N1, Influenza virus A, H1N1; H3N2, Influenza virus A, H3N2; HCOV, Human seasonal coronavirus; HMPV, Human metapeumovirus; HPIV, Human parainfluenza virus; HRSV, Human respiratory syncytial virus; HRV, Human rhinovirus; LPN1, Legionella pneumophila serum type 1; CP, Chlamydia pneumoniae; Li's ALS, Li Lanjuan's artificial liver system (ALS)*.

**Table 2:** Timeline of physical examination and clinical intervention in critically ill patients with COVID-19.

Date*	**Days of follow-up^#^**	**Patient 1**		**Patient 2**	
		**Examination**	**Intervention**	**Examination**	**Intervention**
25-Jan-20	1			Illness onset	
26-Jan-20	2				
27-Jan-20	3				
28-Jan-20	4				
29-Jan-20	5			Confirmed test for SARS-CoV-2	
30-Jan-20	6				
31-Jan-20	7				
1-Feb-20	8				
2-Feb-20	9				
3-Feb-20	10	Illness onset			
4-Feb-20	11				
5-Feb-20	12				
6-Feb-20	13			Admission to hospital; Routine blood test	
7-Feb-20	14				
8-Feb-20	15				
9-Feb-20	16	Confirmed test for SARS-CoV-2			
10-Feb-20	17				
11-Feb-20	18	Admission to hospital; Routine blood test			
12-Feb-20	19				
13-Feb-20	20				Transferred into CCU
14-Feb-20	21				Artificial liver system
15-Feb-20	22				Artificial liver system
16-Feb-20	23				
17-Feb-20	24				Artificial liver system
18-Feb-20	25		Transferred into CCU		
19-Feb-20	26				
20-Feb-20	27				Artificial liver system
21-Feb-20	28				Artificial liver system
22-Feb-20	29				
23-Feb-20	30				
24-Feb-20	31				
25-Feb-20	32				Artificial liver system
26-Feb-20	33				
27-Feb-20	34				
28-Feb-20	35				
29-Feb-20	36				
1-Mar-20	37	PCR of nasopharyngeal swab		PCR of nasopharyngeal swab	
2-Mar-20	38				
3-Mar-20	39	PCR of whole blood; PCR of BALF; PCR of urine; PCR of feces	Bronchoscopy, bronchoalveolar lavage		
4-Mar-20	40	Serological test		PCR of BALF; PCR of urine	Bronchoscopy, bronchoalveolar lavage
5-Mar-20	41				
6-Mar-20	42				
7-Mar-20	43				
8-Mar-20	44				
9-Mar-20	45				
10-Mar-20	46		Thoracic closed chest drainage		
11-Mar-20	47	End of follow-up			
12-Mar-20	48				
13-Mar-20	49				Thoracic closed chest drainage
14-Mar-20	50				
15-Mar-20	51				
16-Mar-20	52				
17-Mar-20	53				
18-Mar-20	54				
19-Mar-20	55				
20-Mar-20	56				
21-Mar-20	57				
22-Mar-20	58			PCR of sputum	
23-Mar-20	59				
24-Mar-20	60				
25-Mar-20	61				
26-Mar-20	62				
27-Mar-20	63			PCR of anal swab	
28-Mar-20	64			PCR of feces	
29-Mar-20	65				
30-Mar-20	66				
31-Mar-20	67				
1-Apr-20	68				
2-Apr-20	69				
3-Apr-20	70				
4-Apr-20	71				
5-Apr-20	72				
6-Apr-20	73				
7-Apr-20	74				
8-Apr-20	75			End of follow-up	

* *Data is expressed as day-month-year*.

# *indicates the period of each event from the first record*.

The study was approved by the ethics committee and the institutional review board of the Renmin Hospital of Wuhan University (file no. WDRY2020-K066).

### Data Collection

Information including clinical symptoms, epidemiological survey, radiological and laboratory results, and clinical treatments were obtained from electronic medical records or via direct communication with the patients and their families. SARS-CoV-2 infection in patients was confirmed by a broad series of investigations including clinical examinations, laboratory tests, chest X-rays, and two independent RT-PCR tests for SARS-CoV-2 using a SARS-CoV-2 ORF1ab/N PCR detection kit (GeneoDx Biotech, Shanghai, China) and a SARS-CoV-2 antibody detection kit (YHLO Biotech, Shenzhen, China). According to the standard procedure protocol, an RT-PCR test was performed for SARS-CoV-2 nucleic acids from nasopharyngeal swabs, BALF samples, feces, urine, whole blood, and sputum. IgM-IgG antibody tests were performed using serum.

Peripheral blood samples were longitudinally collected from two individually confirmed COVID-19 patients and examined for lymphocyte subsets (CD16^+^CD56^+^, CD19^+^, CD3^+^, CD4^+^, and CD8^+^ T cells) by flow cytometry using a flow cytometer BNII (Becton Dickinson, Cockeysville, MD, USA) as previously described (5), the pathological parameter profiles using specific immunoassays, and cytokines using BD FACSCalibur flow cytometer (BD FACSCalibur, BD Bioscience, CA, USA) following manufactures' instructions. Longitudinal plots of parameter data were visualized using GraphPad Prism 7 software (GraphPad Software Inc., San Diego, CA, USA).

### BALF Preparation

The lung segment was locally anesthetized after injection with 2% lidocaine. A total of 150 mL fractions of room-temperature sterile saline were instilled through the bronchial lumen into the right middle or lower lobe of the lung. BALF was retrieved by gentle syringe suction and removed into sterile containers for further analysis.

### Treatments

Both patients received regular medical treatments involving a combination of antiviral, bacterial, and fungal drugs, anti-inflammatory agents, and high-flow oxygen supplements (Table 1). With preexisting comorbidities of hypertension and hematological disorders, both critical COVID-19 patients developed underlying pyemia and underwent interventional therapies including bronchoscopy, bronchoalveolar lavage (on days 29 and 39 post-onset for Patient 1 and Patient 2, respectively), and thoracic closed chest drainage (on days 36 and 48 post-onset for Patient 1 and Patient 2, respectively). In addition, Patient 2 received an artificial liver system treatment six times at 20, 21, 23, 26, 27, and 31 days after the onset of illness, respectively.

## Results

### Clinical Manifestations and Laboratory Findings

Between January 25 and April 8, 2020, two critical COVID-19 patients admitted to the Renmin Hospital of Wuhan University, Wuhan, China, were retrospectively analyzed (Table 1). Both patients (69-year-old female and 43-year-old male) had pre-existing comorbidities of high-grade hypertension and hematological disorders. The main onset symptoms in both cases were cough, asthenia, and dyspnea, whereas one patient (Patient 2) had fever, which is consistent with the clinical signs previously described (2). The chest CT scan in two COVID-19 patients showed typical changes in viral pneumonia, including blurred borders and patchy, scattered ground-glass opacities. After hospital admission, the patients were transferred to the CCU because of the severity of illness.

**Figure 1:**
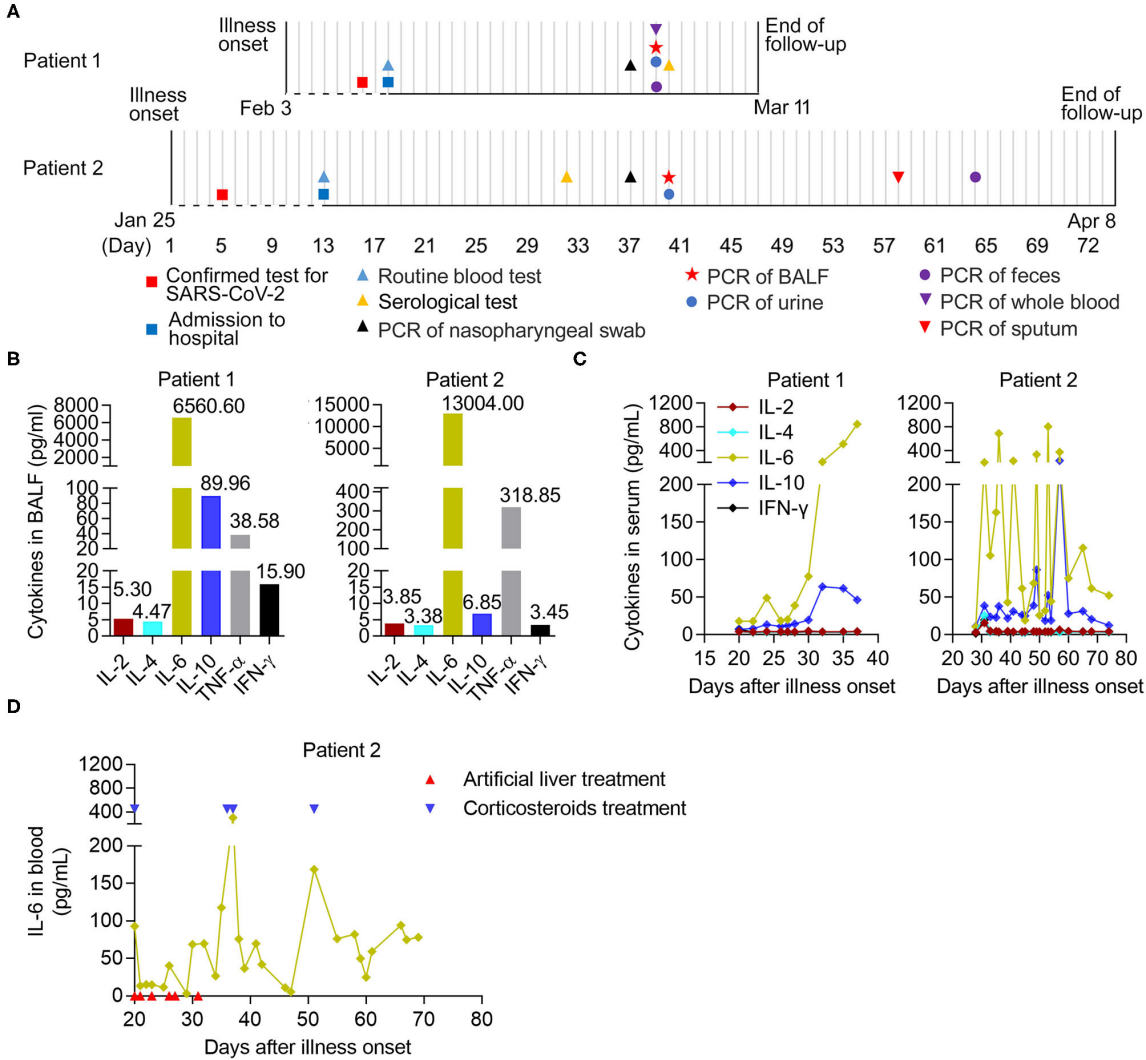
The longitudinal immunological parameters of each critical case with COVID-19. **(A)** Timeline of two critical cases with COVID-19 in hospital after onset of illness. The recorded events of all patients undergoing laboratory tests were marked with different diagrams on the indicated date. BALF, bronchoalveolar lavage fluid. **(B)** The levels of six cytokines in BALF from two critical patients on 29- and 39- days post onsets of Patient 1 and Patient 2, respectively. **(C,D)** The longitudinal immunological and biochemical parameters of each case. **(C)** The levels of six cytokines in serum from patients, normal range (pg/mL): IL-2 (<11.4), IL-4 (<12.9), IL-6 (<20), IL-10 (<5.9), TNF-α (<5.5), and IFN-γ (<18). **(D)** The levels of IL-6 in whole blood from Patient 2. The case received an artificial liver system treatment (indicated by arrow in red) six times at 20, 21, 23, 26, 27, and 31 days post-onset and the corticosteroids therapy (indicated by arrow in blue) at 20, 36, 37, and 51 days post-onset, respectively.

During the hospitalization period, both patients underwent laboratory tests with detailed information in the timeline (Figure 1A). The white blood cell (WBC) count was mildly increased in Patient 1 and normal in Patient 2. Both patients had low cell numbers and ratios of lymphocytes but high numbers of neutrophils (Table 1), which is in line with SARS-CoV-2-induced lymphopenia (6). Increased concentrations of alanine aminotransferase (ALT) and aspartate aminotransferase (AST) in Patient 2 indicated some level of liver dysfunction, as previously reported (14). None of the patients had co-infection with other common respiratory viruses or pathogens (Table 1).

Both nasopharyngeal swab and BALF samples from patients were tested for SARS-CoV-2 RT-PCR with positive results. In addition, the SARS-CoV-2 RT-PCR test results were positive in the urine from Patient 1, along with the sputum and feces from Patient 2, but negative in the whole blood and feces from Patient 1 and the urine from Patient 2. During follow-up, the serum samples from the two patients had noticeably elevated concentrations of SARS-CoV-2 IgM and IgG antibodies (Table 1).

### Treatments and Outcomes

During the period of illness, the two patients underwent medical treatments, including a combination of antiviral, bacterial, and fungal drugs, with anti-inflammatory agents, high-oxygen, and interventional (bronchoscopy, bronchoalveolar lavage, and thoracic closed chest drainage) therapies (Table 1). Importantly, Patient 2 received Li's artificial liver system treatment six times in succession (20, 21, 23, 26, 27, and 31 days after onset of illness) (Table 2), aiming to reduce the potential lung injury resulting from the cytokine storm (15, 16). Later, the outcomes resulted in the death of Patient 1 and recovery of Patient 2, respectively.

### Analysis of Immunological and Biochemical Parameters in Critical COVID-19 Patients

We further analyzed the dynamic immunological and biochemical parameters of the two critical cases. To directly evaluate inflammation in the lungs, the levels of six cytokines (IL-2, IL-4, IL-6, IL-10, TNF-α, and IFN-γ) in BALF samples from two critical patients (29 and 39 days after onset for Patient 1 and Patient 2, respectively), were measured upon admission and during treatment. Among them, IL-6 and TNF-α showed significant increases compared to normal levels. Notably, increased secretion of the anti-inflammatory cytokine IL-10 was also observed (Figure 1B). Furthermore, we combined the longitudinal immunological and biochemical data of each case and plotted their fluctuation patterns against the time points post-onset. During treatment, the levels of IL-6 and IL-10 were continuously elevated in the serum of Patient 1. In the serum from Patient 2, these two cytokine levels dropped transiently due to the targeted treatment and remained high in the early stage; however, these sharply declined later on (after 57 days post-onset) (Figure 1C). Particularly, the levels of IL-6 in whole blood from Patient 2 transiently decreased during artificial liver treatment (21 to 31 days post-onset) and sharply declined after 37 days post-onset, indicating the beneficial effect from artificial liver treatment (Figure 1D).

### High Risk and Longitudinal Factors Associated With Cytokine Overproduction in Critical COVID-19 Patients

**Figure 2:**
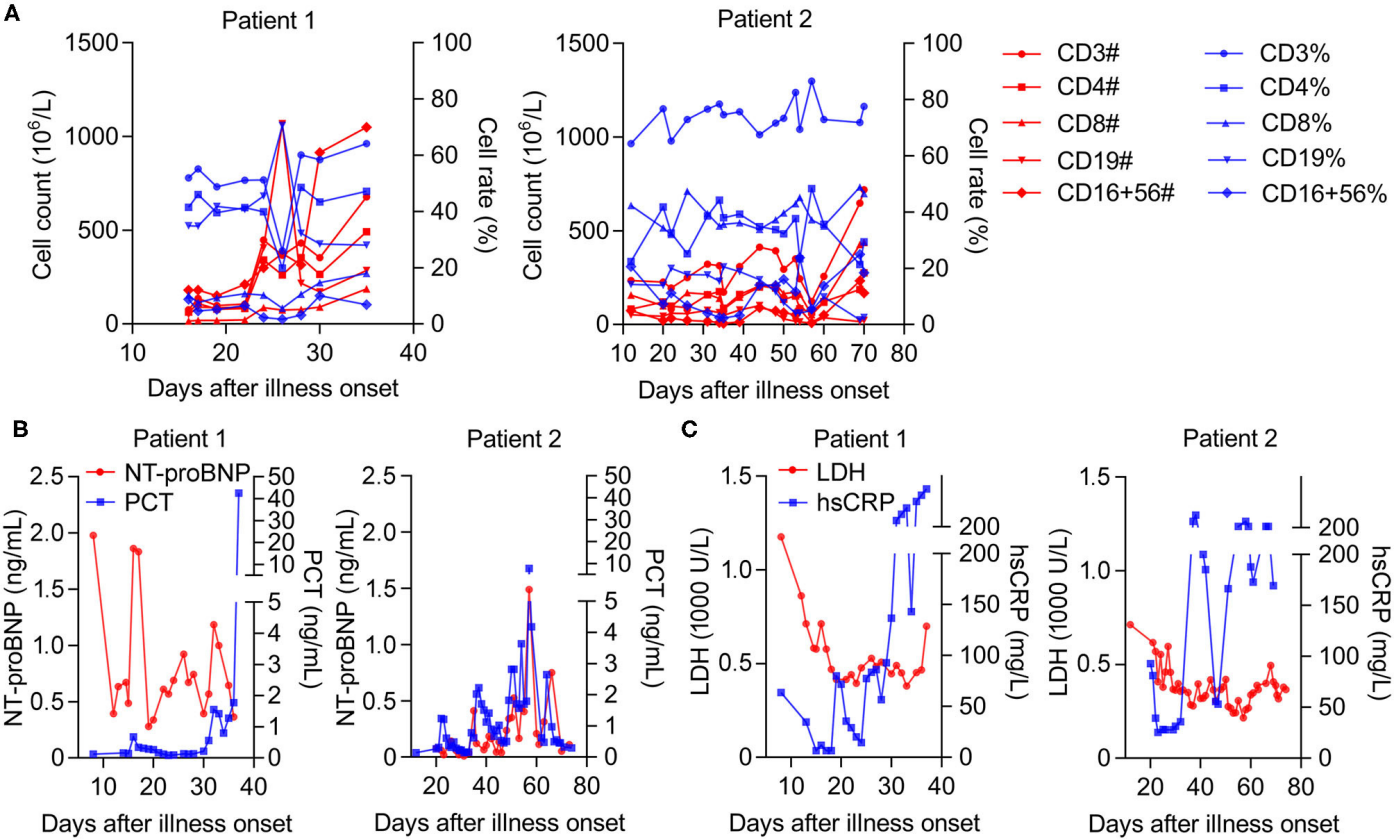
The longitudinal biochemical parameters of each critical case with COVID-19. **(A)** The number and ratio of T cell populations in whole blood from patients, normal range (count, 10^6^/L; ratio, %): CD16^+^CD56^+^ (84–724; 5–26), CD19^+^ (80–616; 5–22), CD3^+^ (723–2,737; 56–86), CD4^+^ (404–1,612; 33–58), and CD8^+^ (220–1129; 13–39). **(B)** The levels of NT-proBNP (N terminal pro B type natriuretic peptide) in plasma or PCT (Procalcitonin) in serum from patients, normal range (ng/mL): NT-proBNP (0–0.9); PCT (<0.1). **(C)** The levels of LDH (Lactate dehydrogenase) in serum or hsCRP (high-sensitivity C-reactive protein) in whole blood from patients, normal range: LDH (120–250 U/L); hsCRP (0–3 mg/L).

Considering that decreased T cell populations contribute to the severity of COVID-19 (6), a detailed analysis of lymphocytopenia subtypes revealed that the number rather than the ratio of CD16^+^CD56^+^, CD3^+^, and CD4^+^ T cells decreased more significantly than that of CD19^+^ and CD8^+^ T cells in both cases in the early stage. However, these were obviously restored later (after 30- and 57-days post-onset in Patients 1 and 2, respectively) (Figure 2A). Correspondingly, we observed a successively increased level of PCT (procalcitonin) in Patient 1; however, Patient 2's PCT levels were high in the early stage but sharply declined later (57 days post-onset) (Figure 2B), suggesting a good prognosis from MOF in Patient 2 but not in Patient 1. We also noticed a decreased level of N-terminal pro-B-type natriuretic peptide levels in both cases (Figure 2B), indicating a certain relief from acute cardiovascular failure. More importantly, there was a successive increase in high-sensitivity C-reactive protein (hsCRP), with a high level of lactate dehydrogenase (LDH) in Patient 1. Meanwhile, these high factor levels rapidly reduced at the late treatment stage in Patient 2 (after 67 days post-onset) (Figure 2C), indicating the deterioration of Patient 1 and improvement of Patient 2 in terms of their COVID-19 progression.

## Discussion

Our analysis of two typically treated patients with critical COVID-19 explores the further discovery of the high risk of poor outcomes related to cytokine overproduction in severe COVID-19 via the detection of hyperinflammation in BALF. Among multiple cytokines (including IL-2, IL-4, IL-6, IL-10, TNF-α, and IFN-γ) contributing to an excessive inflammatory response in lung injury (17), we observed a dominantly elevated level of IL-6 in BALF samples from both critical COVID-19 cases. Although exuberant plasmacytosis and some transcriptional changes of inflammatory genes in the BAL specimens from COVID-19 patients suggest local hyperinflammation in the lungs (4, 10–12), these provides direct evidence that the overactive production of cytokines (especially IL-6) occurred upon SARS-CoV-2 infection in the lungs and led to the subsequent development of a cytokine storm in the blood, which might act as a prognostic indicator for the severity of COVID-19.

To extend the relationship between host immune response and hyperinflammation detected from BALF samples, the longitudinal characteristics of cytokine profiles, lymphocyte responses, and pathological parameters were further analyzed in the peripheral blood of critical COVID-19 patients. During treatment, the levels of IL-6 were continuously elevated in the serum of Patient 1 and remained high in the early stage. Notably, Patient 2 received targeted treatments and their IL-6 levels dropped transiently after six rounds of artificial liver treatment in the early stage (21–31 days post-onset), until they sharply declined after 37 days post-onset. This decline could be explained by the use of corticosteroids (18), which temporarily inhibits cytokine overproduction (especially IL-6) and is further stably relieved with the application of Li's artificial liver system (16). We also noticed increased secretion of the anti-inflammatory cytokine IL-10, indicating divergence in the immunological lung injury, as previously observed (17). It could be recommended that the decreased levels of IL-6 and IL-10 in blood from COVID-19 patients act as the sign of the alleviating disease, which might be helpful for personal physical examination in the future clinical guidelines. Interestingly, a similar phenomenon was also observed in lymphocytopenia subtype population changes and PCT levels. Notably, the number of CD16^+^CD56^+^, CD3^+^, and CD4^+^T cells decreased more significantly than that of CD19^+^ and CD8^+^ T cells, which is in line with the findings of reduced T cell populations as well as lymphopenia leading to the severity of COVID-19 (5, 6, 19). Importantly, the changes in PCT levels in the two cases were correlated with the immune responses, reflecting the status of pyemia-related MOF in critical COVID-19 patients (20).

Emerging biomarkers have been identified as prognostic factors for severe COVID-19. In addition to lymphocytes, high levels of LDH, and hsCRP can predict interpretable mortality in COVID-19 patients (21). Based on this, the longitudinal levels of LDH and hsCRP in the peripheral blood of critical COVID-19 patients were also retrospectively analyzed. A sequentially increased level of hsCRP along with a high level of LDH in Patient 1 was reported, whereas these high levels were rapidly reduced in the late treatment stage in Patient 2. These changes demonstrated deterioration of Patient 1 and improvement in Patient 2 in terms of their COVID-19 progression resulting from a cytokine storm during treatment.

Until now, the common strategy for treating COVID-19 is a host-based treatment, including anti-infection, anti-inflammatory, or anti-cytokine overactivity therapies (22, 23). In our retrospective study, we summarized the treatments for two severe COVID-19 patients, such as the combination of antiviral, bacterial, and fungal drugs, along with anti-inflammatory agents and high-flow oxygen supplementation as previously described (24). Due to preexisting comorbidities of hypertension and hematological disorders, both critical COVID-19 patients developed underlying pyemia, which frequently reemerges in COVID-19 patients in ICUs (14). Both underwent interventional therapies including bronchoscopy, bronchoalveolar lavage, and thoracic closed chest drainage. In particular, Patient 2 received Li's artificial liver system treatment six times, ranging from 20 to 31 days after onset. Interestingly, we reviewed the levels of cytokines and pathological parameters in Patient 2 and found a visible restoration at the late stage of treatment (after 57 days post-onset), which may have resulted in the good outcome in Patient 2. Commonly, the time period of SARS virus-associated ARDS is 16.52 days post-onset (25), while the duration of other pulmonary and extrapulmonary ARDS is more than 60 days (26), however, during the treatment, the duration of COVID-19 ARDS is up to 90 days post-onset (27). It could be view as a normal period of treatment in this observational study. Consequently, except for targeted therapies against virus or other pathogen infections such as organ function support, artificial liver systems and facilitating the removal of cytokines through blood purification technology (19, 28) may be beneficial for the recovery of critical COVID-19 patients.

The limitations of this study include its small sample size and retrospective design. Some considerations should be considered when interpreting the findings. First, the BALF samples from the enrolled patients were difficult to collect, and we could not determine the longitudinal presence of SARS-CoV-2 and the dynamic levels of cytokines, such as daily collected samples. Second, more evidence should be obtained in order to investigate the relationship between the host immune response and hyperinflammation (such as IL-6 production) detected in BALF. Third, to exclude the individual variation including age and gender, a larger sample size might statistically support the conclusion that the removal of cytokines through Li's artificial liver system was beneficial for the recovery of critical COVID-19 patients. Finally, the data of longitudinal cytokine levels in the early stage during artificial liver treatment were absent, leading to failure to directly access the decrease of cytokine levels after the immediate performance of treatment. Fortunately, we presented the longitudinal level of IL-6 in whole blood from Patient 2 transiently decreased during first five rounds of treatment (21–27 days post-onset), indicating the beneficial effect from artificial liver treatment. Nevertheless, we believe this report could provide an appropriate reference for artificial liver treatment in future large-scale clinical trials for critically ill COVID-19 patients.

Collectively, we reported that the clinical characteristics of two critically ill COVID-19 patients were similar to those reported previously. We also revealed the poor outcomes related to cytokine overproduction in critical COVID-19 patients with hyperinflammation detected in BALF and the potential effect on the decrease of cytokines through an artificial liver system is beneficial for the treatment of critical COVID-19 patients. In sum, this single uncontrolled clinical observation is compatible with a possible effect of the artificial liver intervention in reducing circulating cytokine levels, and suggests that the supportive intervention ought to be further tested in adequately designed and powered clinical trials.

## Data Availability Statement

The original contributions presented in the study are included in the article/supplementary material, further inquiries can be directed to the corresponding author/s.

## Ethics Statement

The studies involving human participants were reviewed and approved by the Ethics Committee and the Institutional Review Board of the Renmin Hospital of Wuhan University (file no. WDRY2020-K066). The patients/participants provided their written informed consent to participate in this study. Written informed consent was obtained from the individual(s) for the publication of any potentially identifiable images or data included in this article.

## Author Contributions

ZL, CZ, YX, and JW: conceptualization and investigation. ZR, XC, MS, and PP: methodology and software. CZ, XC, JH, FW, and HS: resources and data curation. ZL and CZ: validation and writing. ZR, XC, MS, PP, JH, FW, and HS: formal analysis. ZL, CZ, and YX: funding acquisition. YX and JW: supervision and writing—review & editing. All authors have approved to submit manuscript of this study and took responsibility for the integrity of the data and the accuracy of the data analysis.

## Funding

This study was supported by the National Natural Science Foundation of China (81971936 and 82041004 to YX, 81672079 to CZ, 31800147 and 32070148 to ZL) and the Guangdong Basic and Applied Basic Research Foundation (2019A1515011073 to ZL). The sponsors had no role in the design, execution, interpretation, or writing of the study.

## Conflict of Interest

The authors declare that the research was conducted in the absence of any commercial or financial relationships that could be construed as a potential conflict of interest.

## Publisher's Note

All claims expressed in this article are solely those of the authors and do not necessarily represent those of their affiliated organizations, or those of the publisher, the editors and the reviewers. Any product that may be evaluated in this article, or claim that may be made by its manufacturer, is not guaranteed or endorsed by the publisher.
